# The Medical Profession in Ancient Times

**Published:** 1856-08

**Authors:** John Watson

**Affiliations:** Surgeon to the New York Hospital


*The Medical Profession in Ancient Times.* An Anniversary Discourse delivered before the New York Academy of Medicine, November 7, 1855. By JOHN WATSON, M. D., Surgeon to the New York Hospital. (Published by order of the Academy.)

We are indebted to the author for three copies of this beautiful address. One of these may be found in the library of the County Medical Society, and another in the library of the Young Men's Association of this city, where they have been placed by the liberality of the distinguished author.

In the preparation of this discourse, Dr. Watson has evinced more than usual industry and scholarship. It is not the old re-hash of Hippocrates, Galen and Celsus, with which we are so often entertained in introductory lectures; but a learned and thorough review of medical history, not only in the apocryphal days of its origin, but in those later days when it had become an organized profession. The causes which led to the ancient division into Methodists and Empirics — a division existing at the present day as clearly as then — are ably discussed and treated. We regret that we have not space to do justice to Dr. Watson's labors, but can only say that we are delighted with their result, and cordially commend it to those members of the profession who may have access to it.

